# Deep learning for cancer type classification and driver gene identification

**DOI:** 10.1186/s12859-021-04400-4

**Published:** 2021-10-25

**Authors:** Zexian Zeng, Chengsheng Mao, Andy Vo, Xiaoyu Li, Janna Ore Nugent, Seema A. Khan, Susan E. Clare, Yuan Luo

**Affiliations:** 1 Department of Preventive Medicine, Feinberg School of Medicine, Northwestern University, 750 N Lake Shore Drive Room 11-189, Chicago, IL 60611 USA; 2 Department of Data Sciences, Dana-Farber Cancer Institute, Harvard T.H. Chan School of Public Health, Boston, MA USA; 3 Committee on Developmental Biology and Regenerative Medicine, The University of Chicago, Chicago, IL USA; 4 Tsinghua University, Beijing, China; 5 Research Computing Services, Northwestern University, Chicago, IL USA; 6 Department of Surgery, Feinberg School of Medicine, Northwestern University, NMH/Prentice Women’s Hospital Room 4-420 250 E Superior, Chicago, IL 60611 USA; 7 Department of Surgery, Feinberg School of Medicine, Northwestern University, Robert H Lurie Medical Research Center Room 4-113 250 E Superior, Chicago, IL 60611 USA

**Keywords:** Convolutional neural network, Cancer, Classification, Whole-exome sequencing, Somatic mutation, Germline variants

## Abstract

**Background:**

Genetic information is becoming more readily available and is increasingly being used to predict patient cancer types as well as their subtypes. Most classification methods thus far utilize somatic mutations as independent features for classification and are limited by study power. We aim to develop a novel method to effectively explore the landscape of genetic variants, including germline variants, and small insertions and deletions for cancer type prediction.

**Results:**

We proposed DeepCues, a deep learning model that utilizes convolutional neural networks to unbiasedly derive features from raw cancer DNA sequencing data for disease classification and relevant gene discovery. Using raw whole-exome sequencing as features, germline variants and somatic mutations, including insertions and deletions, were interactively amalgamated for feature generation and cancer prediction. We applied DeepCues to a dataset from TCGA to classify seven different types of major cancers and obtained an overall accuracy of 77.6%. We compared DeepCues to conventional methods and demonstrated a significant overall improvement (*p* < 0.001). Strikingly, using DeepCues, the top 20 breast cancer relevant genes we have identified, had a 40% overlap with the top 20 known breast cancer driver genes.

**Conclusion:**

Our results support DeepCues as a novel method to improve the representational resolution of DNA sequencings and its power in deriving features from raw sequences for cancer type prediction, as well as discovering new cancer relevant genes.

**Supplementary Information:**

The online version contains supplementary material available at 10.1186/s12859-021-04400-4.

## Background

The majority of cancer driver gene studies have been focusing on the identification of individual somatic point mutations [1, 2]. However, somatic mutations are often highly heterogeneous between cancer genomes, even within the same type of cancer, and only represent for a small portion of the genome variations [3]. While many methods attempted to address the complex mutational heterogeneity in cancer, driver gene identification still remains a challenge due to the limited capability in integrating other genome components for integrative study [4–8]. Other genome components, such as nonsense mutations of insertions and deletions, as well as germline variation, were largely ignored in the past but have been recently highlighted to play a significant role for cancer development [9–11]. Genome components, including somatic mutations, germline variants, insertions and deletions, when studied together, especially in an interactive term, give rise to the challenges of model complexity and study power [12].

Due to the limitation of analysis power, methods including Bayesian classifier [13], regression models [14, 15], and KNN [16] are not optimal in handling such high-dimensional features interactively. To circumvent these challenges, labor intensive feature engineering using prior knowledge need to be performed prior to modeling [17]. These conventional learning algorithms rely heavily on data representations and are typically designed by domain experts. The complexity of the human genome and the amount of required human effort make it difficult to derive meaningful features [18, 19], whereas deep learning can automatically learn a good feature representation [20]. Deep learning has recently emerged with the advances in big data with the power of parallel computing and sophisticated algorithms. Furthermore, deep learning models are exponentially more efficient than conventional models in learning intricate patterns from high-dimensional raw data with little guidance [20–23]. Typically, convolutional neural networks (CNNs) computes convolution on small regions by sharing parameters between regions [24], which allows training models on large DNA sequences. Recent examples of exploring the application of CNNs within raw sequencing data include DeepBind [25], DanQ [26], DeepSEA [27], DeepCpG [28].

Inspired by the successful applications of deep learning models in genomics data, and in an attempt to study somatic mutations, germline variants, insertions and deletions collectively and interactively, we propose to utilize deep learning models to study the tumor raw sequences, namely Deep learning for disease Classification using exome sequencings (DeepCues). Specifically, we propose to use a CNNs model to utilize tumor raw DNA sequences for cancer type classification and more importantly, relevant gene identification. In addition to raw tumor sequence, we also investigated the utility of germline DNA sequences. Collectively, we have identified a subset of genes that are relevant for each cancer development. In a pilot study utilizing 4174 samples across seven major cancer types from The Cancer Genome Atlas (TCGA), we were able to achieve an accuracy of 77.6% in predicting cancer types using the raw tumor sequences. Germline variants dominant somatic mutations number-wise, strikingly, in the attempt of utilizing germline raw sequences only, we were able to achieve an accuracy of 73.9%. Using the trained models, we have identified several known cancer driver genes, along with a list of genes that have not been previously reported as cancer driver genes.

## Results

The following cancers were analyzed: brain cancer, breast cancer, colorectal cancer, kidney cancer, lung cancer, prostate cancer, and uterus cancer. Germline and somatic mutations from 4174 samples across seven major cancer types were obtained from the TCGA [29]. To construct raw sequences for each cancer sample, we merged the reference genome sequence with the identified germline variants and somatic mutations, individually or in combination. To prepare the germline variants, aligned sequencing data derived from blood or adjacent normal tissues were recalibrated, and variants were called using HaplotypeCaller from GATK package [30]. SnpEFF was used for functional annotation [31], and variants annotated with moderate effects were missense mutations and in-frame shifts; nonsense mutations were annotated as high effects. In parallel, somatic mutations for the matched samples were obtained directly from TCGA and the same functional annotation processes were performed. In total, 4600 virtual machines were utilized for 119,000 CPU hours for these tasks. Overall, we identified 45,119,052 germline variants and 957,115 somatic mutations from the 4174 matched samples (Table 1).

**Table 1:** The number of samples of each cancer and the corresponding number of germline variants and somatic mutations

Cancer	Cancer #	Blood #	Adjacent normal #	Germline moderate	Germline high	Somatic moderate	Somatic high
Breast cancer	959	936	23	10,911 (71)	641 (5)	65 (10)	16 (3)
Colorectal cancer	420	395	25	10,331 (241)	621 (15)	293 (79)	81 (18)
Brain cancer	763	763	0	9572 (55)	550 (3)	94 (30)	23 (6)
Uterus cancer	530	507	23	10,894 (103)	650 (7)	645 (152)	160 (28)
Lung cancer	730	713	17	9717 (37)	555 (2)	217 (15)	43 (3)
Kidney cancer	332	332	0	10,882 (124)	634 (8)	51 (6)	14 (1)
Prostate cancer	440	440	0	9744 (53)	558 (4)	34 (22)	7 (3)

Variants annotated with moderate effects are defined as missense mutations and in-frame shifts; variants annotated as high effects are defined as nonsense mutations

*Number in parenthesis is standard deviation (SD)

**Fig. 1:**
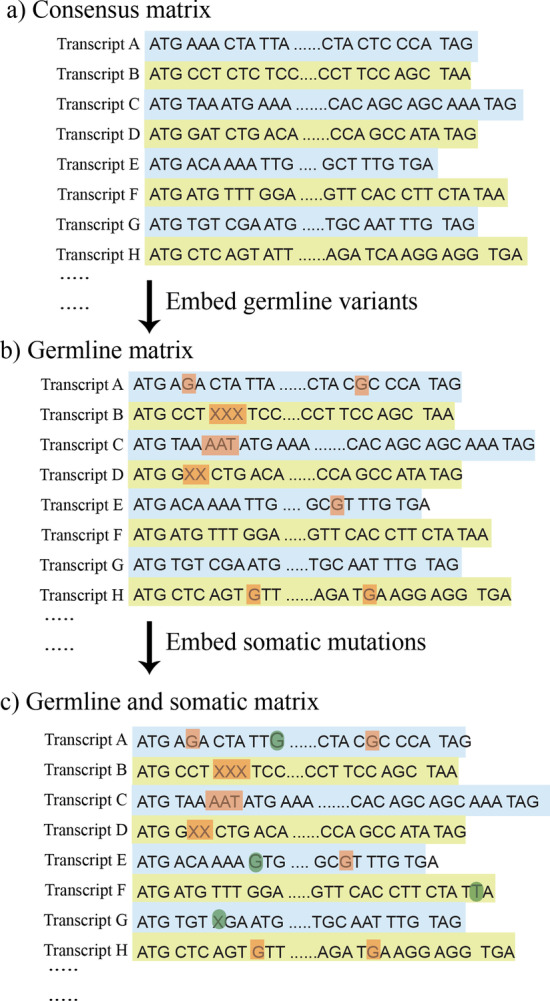
Feature generation for proposed models. **a** The transcript sequences were retrieved from RefSeq and were formed as a consensus matrix. **b** Each patient’s germline variants were embedded in the consensus matrix, forming a germline raw sequence for each sample. The brown dots are the germline variants including polymorphisms, deletions, and insertions. As an illustration, single nucleotide polymorphisms were identified and embedded in transcript A, E, and H. An in-frame shift deletion was embedded in transcript B and an in-frame shift insertion was embedded in transcript C. A frame shift deletion and frame shift insertion is embedded in transcripts D and E, respectively. Transcript F and G remained the same. **c** Each patient’s somatic mutations were embedded in the germline raw sequence (from B), forming a germline and cancer raw sequences. The green dots are the somatic mutations including SNVs, insertions, and deletions. As an illustration, the tissue gained somatic mutations in transcript A and E; gained a stop loss in transcript F; and gained a deletion that shifted the frame in transcript G

As a pilot study, we derived features only from genes that have been implicated in cancers using a list of 719 consensus genes (Additional file 1: Table S1) from the Catalogue of Somatic Mutations in Cancer (COSMIC), which is a mutation catalogue with comprehensive mutation information curated from about 542,000 tumor samples [32]. In our dataset, these consensus genes corresponded to 985 canonical transcripts (Additional file 2: Table S2) and thus, the transcripts were used to train and evaluate our proposed models. To construct raw sequences for each sample, sequences in RefSeq database was started as references (Fig. 1a). The RefSeq database was named as consensus matrix. This consensus matrix consists of 24,286 transcripts. The average length of these sequences were 3375 bases. For each individual, the identified germline variants were constructed into the consensus matrix, forming a germline raw sequence (Fig. 1b). Once a germline raw sequence was formed for each sample, somatic mutations were then constructed in the germline raw sequence, forming a cancer raw sequence (Fig. 1c). It has been suggested that mutations prefer certain codons and the distance between amino acid changes have been described [33]. Moreover, the position within the codon where the mutation occurs is critical. To incorporate codon information into our model features, one hot encoding was applied with every three nucleotides and was encoded as a binary unit. The combination of four nucleotides (A, C, T, and G) results in a vector with 64 dimensions to represent each codon combination.

**Fig. 2:**
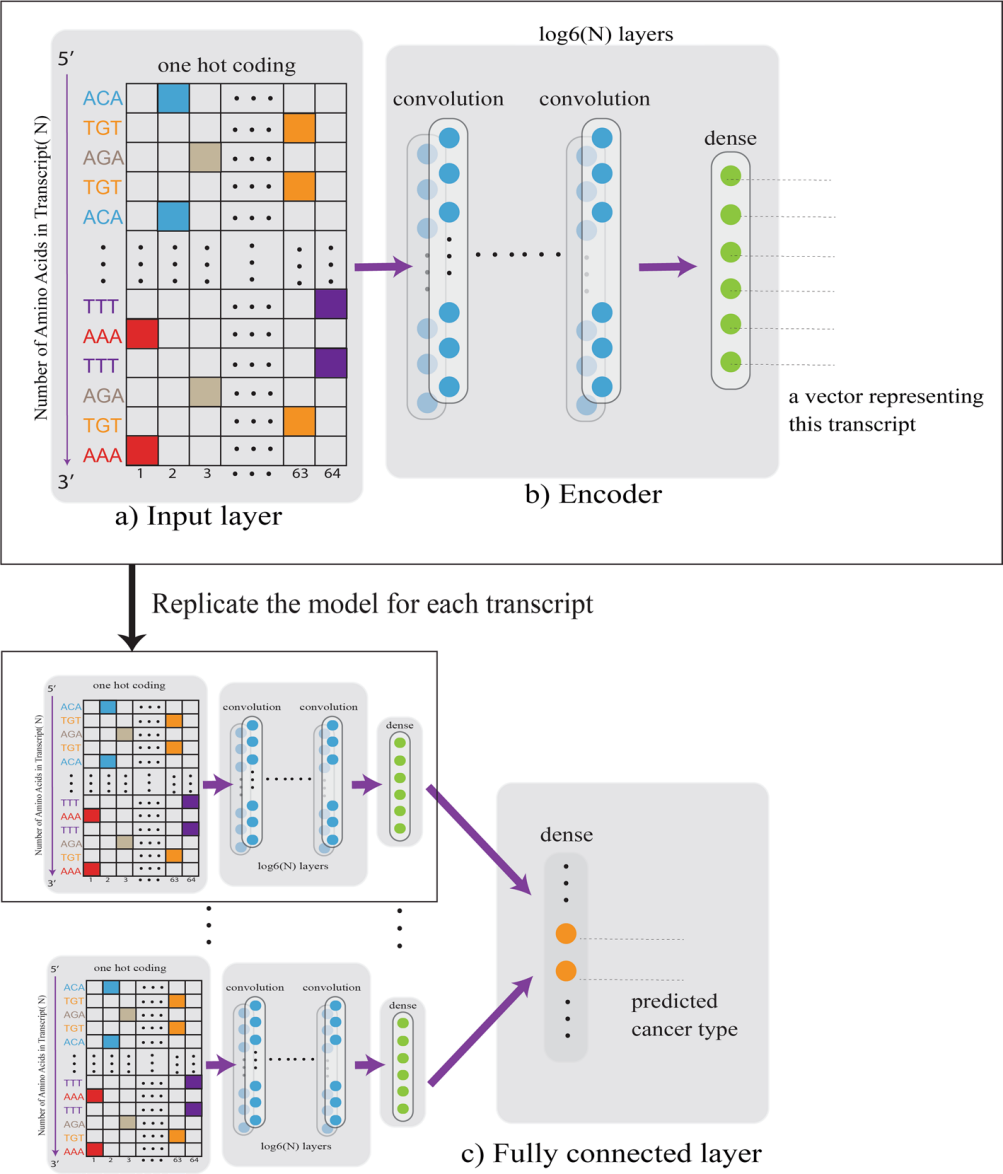
The architecture of the convolutional neural network. Component **a** is the input layer with one hot encoding with the column number equals 64 (number of total possible codons) and the row number equals the number of codons in the transcript. Component **b** is the encoder component containing a sequence of layers, each consisting of a convolutional layer, followed by a Leaky Rectified Linear Unit and average pooling layer. The number of convolution layers is determined by the gene length. Component **c** is a fully connected layer that combined all the outputs from the component **b** and has k outputs for k diseases

**Fig. 3:**
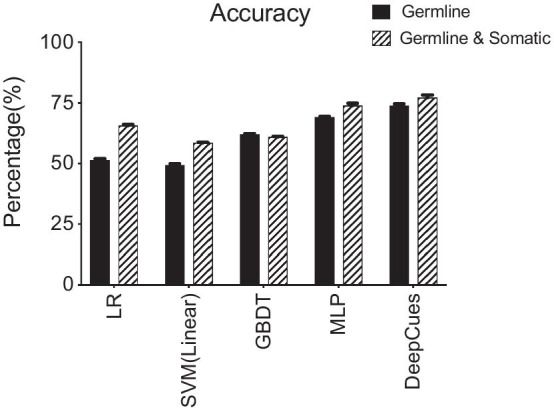
Prediction accuracy comparisons between DeepCues and baseline models. The compared methods include penalized logistic regression (LR) and support vector machine (SVM) with linear kernel, Gradient Boosting Decision Tree (GBDT), and Multiple Layer Perceptron (MLP) model

A convolutional framework that consists of multiple layers was used in our study (Fig. 2). The framework has three components: input layer (Fig. 2a), encoder layer (Fig. 2b) (multiple convolutional and dense layers), and fully connected layer (Fig. 2c). We first trained convolutional neural networks (CNNs) using the 985 pathogenetic transcripts and calculated overall classification accuracy for each cancer type. Using only the germline raw sequence as input (Method), we achieved an overall accuracy of 73.9% (SE = 0.7%) (SE standard error). Using the cancer raw sequence as input, the achieved overall accuracy was 77.6% (SE = 0.9%). To compare our method to other conventional cancer classification methods and to benchmark our results, we calculated baseline accuracies using logistic penalized linear regression and linear SVM, which are among the most widely utilized methods for cancer classification. We also evaluated more advanced models including Gradient Boosting Decision Tree (GBDT) and Multiple Layer Perceptron (MLP). Logistic penalized linear regression resulted in an overall accuracy of 51.5% (SE = 0.5%) and 65.5% (SE = 0.3%) using the germline and cancer data, respectively; linear SVM yielded an overall accuracy of 49.4% (SE = 0.4%) and 58.6% (SE = 0.3%). Likewise, Gradient Boosting Decision Tree (GBDT) achieved an overall accuracy of 62.1% (SE = 0.24%) and 61% (SE = 0.21%) (Fig. 3). Using sequence data as input, MLP model achieved an overall accuracy of 69.2% (SE = 0.23) and 74% (SE = 0.89). Using the same input information, our proposed method significantly outperformed the conventional methods (*p* < 0.001).

For each cancer type, classification precision, recall, and f-measure were characterized (Table 2). Of note, multiclass data will be treated as if binarized under a one-vs-rest transformation. Using only germline raw sequence, we found breast cancer and colorectal cancer yielded the highest F-measure scores. Using tumor raw sequence data, we found breast cancer, colorectal cancer, and brain cancer had the highest F-measure scores. Multilabel confusion matrix averaged between the 10 runs were used to evaluate the effectiveness of our proposed method (Table 3). In our proposed model, tumor raw sequence is a combination of somatic mutations and germline raw sequences. Adding the somatic mutation data increased F-measures for breast cancer, brain cancer, and uterus cancer significantly (*p* = 6.7E−03, 3.8E−06, and 1.9E−02 respectively).

**Table 2:** Precision and recall for our proposed model

	Germline sequence			Cancer sequence		
	Precision	Recall	F-measure	Precision	Recall	F-measure
Breast	81.9% (2.7%)	83.4% (4.2%)	81.4% (1.8%)	85.6% (2.0%)	90.7% (1.8%)	**87.8% (1.1%)**
Colorectal	85.9% (1.9%)	83.9% (2.1%)	84.6% (0.8%)	84.7% (4.5%)	87.1% (2.6%)	84.7% (2.1%)
Brain	73.0% (1.5%)	66.5% (4.3%)	68.5% (1.9%)	**87.5% (2.7%)**	**78.4% (2.0%)**	**82.2% (0.9%)**
Uterus	76.3% (4.9%)	62.7% (6.9%)	64.2% (3.9%)	85.3% (1.8%)	68.2% (3.3%)	**75.1% (1.7%)**
Lung	64.8% (3.2%)	75.5% (4.7%)	67.7% (1.5%)	70.3% (4.9%)	78.4% (5.5%)	71.2% (2.0%)
Kidney	76.9% (2.5%)	71.5% (2.8%)	73.4% (1.1%)	77.6% (4.7%)	68.7% (5.9%)	69.5% (2.3%)
Prostate	70.8% (4.2%)	55.7% (6.0%)	58.9% (3.2%)	65.6% (5.5%)	50.5% (9.7%)	49.1% (5.9%)

The experiment is replicated for 10 times and the number in parenthesis is standard error. The bolded number are those that significantly improved in cancer sequence compared to germline sequence

**Table 3:** The confusion matrix for our proposed model

Predicted	Predicted class cancer sequence			
	Germline sequence		Cancer sequence	
True	Positive	Negative	Positive	Negative
Breast				
Positive	161	31	169	22
Negative	36	608	39	604
Colorectal				
Positive	73	11	72	11
Negative	15	737	8	743
Brain				
Positive	124	29	100	53
Negative	67	616	53	628
Uterus				
Positive	64	42	62	44
Negative	22	708	16	713
Lung				
Positive	88	58	94	52
Negative	33	657	60	628
Kidney				
Positive	48	19	49	19
Negative	18	751	26	741
Prostate				
Positive	57	31	50	38
Negative	30	719	36	710

The number is average of the 10 runs for test set prediction

In an attempt to identify novel cancer driver genes, we integrated an additional 985 transcripts to our current feature pools. When using only the germline raw sequence as input features, we achieved an overall accuracy of 82.7% (SE = 0.6%). Using the raw cancer sequence as input, we achieved an overall accuracy of 80.0% (SE = 0.9%). Similarly, for each type of cancer, we calculated precision, recall, and F-measure using either the germline raw sequence or the cancer raw sequence (Table 4). Using only germline data, we found breast cancer and colorectal cancer had the highest F-measure scores. Using both germline and somatic mutation data, we found breast cancer, colorectal, and uterus cancer had the highest F-measure scores. Consistently, best performances were found within breast cancer and colorectal cancer in both models.

**Table 4:** Precision and recall for our proposed model

	Germline sequence			Cancer sequence		
	Precision	Recall	F-measure	Precision	Recall	F-measure
Breast	87.1% (2.8%)	91.9% (1.6%)	89.0% (1.2%)	89.1% (1.6%)	87.2% (2.2%)	87.8% (0.7%)
Colorectal	87.8% (1.7%)	94.0% (1.6%)	90.6% (0.8%)	86.1% (3.8%)	90.8% (2.9%)	87.5% (2.2%)
Uterus	90.0% (1.8%)	72.8% (6.8%)	78.0% (5.5%)	84.9% (3.1%)	71.4% (2.8%)	76.6% (1.0%)
Brain	88.3% (4.4%)	58.6% (5.2%)	67.9% (2.2%)	77.3% (2.5%)	**75.9% (3.5%)**	**75.6% (1.5%)**
Lung	69.1% (4.9%)	79.9% (7.2%)	69.8% (2.7%)	73.7% (3.1%)	75.6% (4.0%)	73.4% (1.8%)
Kidney	81.9% (2.7%)	81.3% (2.0%)	81.3% (1.5%)	70.0% (3.8%)	76.0% (5.0%)	71.2% (3.3%)
Prostate	76.4% (6.1%)	67.3% (7.2%)	66.0% (3.7%)	72.2% (2.7%)	65.5% (3.5%)	68.1% (2.3%)

The experiment is replicated for 10 times and the number in parenthesis standard error. The bolded number are those that significantly improved in cancer sequence compared to germline sequence

Using the coefficients derived from the fully connected layer, the model can be extended to prioritize genes that are relevant for each cancer type. The analysis was repeated 10 times with different initial seeds and top 20 genes were labeled in each replicate. The studied genes were subsequently ranked by frequency among all replicates. Top ranked genes were considered as cancer relevant genes and were summarized in Additional files 4–7: Table S4–S7. As a result of breast cancer relevant gene discovery, strikingly, 8 of the top 20 genes overlapped with the COSMIC breast cancer top 20 genes when we use cancer raw sequences as input. The high consensus rate (40%) partially validated that our method was effective in identifying cancer relevant genes. In addition, we have identified cancer relevant genes that have not been previously explored for breast cancer (Table 5).

**Table 5:** The top 20 genes relevant genes with breast cancer derived from the 985 pathogenetic transcripts and the 1970 transcripts

985 transcripts		1970 transcripts	
Germline	Cancer	Germline	Cancer
**TCF3**	**GATA3**	FOXP1	CASP8
**FOXP1**	APC	TCF3	PALB2
**LEF1**	**RNF213**	**SPATA31A3**	**RXFP3**
**PAFAH1B2**	**PIK3CA**	PAFAH1B2	**DOCK2**
**BLM**	PPP2R1A	CCNE1	ANK1
**PPFIBP1**	**CDH1**	**ADAM23**	**ADAM23**
**PALB2**	CHD2	NCOA4	ITK
**MUC4**	**RUNX1**	**KC6**	TCF3
**CCDC6**	MSH6	PALB2	CCNE1
**LIFR**	CNBD1	**AMOT**	FOXP1
**MITF**	**KMT2C**	LEF1	**ABHD13**
**CCNE1**	**ARID1A**	**CPEB2**	**SPATA31A3**
**ZCCHC8**	TBX3	**SPATA31D1**	**XPO6**
TP53	KRAS	**IL21**	**FRYL**
**FOXO4**	CHD4	BLM	**RFC5**
**EBF1**	**KMT2D**	ITK	SDHAF2
**TERT**	NRAS	ZNF507	**POU4F1**
**GMPS**	MAP3K1	**CCDC6**	PICALM
**CCNC**	HLA-A	TERT	LEF1

The bold genes in the 985 transcripts are the ones found in COSMIC top 20 genes. The bold genes in the 1970 transcripts are the ones in the unknown transcripts

## Discussion

The development of high throughput sequencing technology has enabled the cataloging of large-scale genetic information. To help improve cancer diagnosis and targeted therapies, cancer type classification methods are continually being upgraded. Traditionally, the majority of classification methods based on DNA sequencing data has relied on studying single point somatic mutations with various regression models [34–36]. Mutations involving insertions and deletions as well as germline mutations have been largely ignored due to the high dimensionality problem. Given that many methods are already limited in their ability to study so many variables, it has been even more challenging to integrate these variables and study them interactively. To deal with these challenges, groups have proposed aggregating mutations on a gene level to be studied as a feature [35, 37–39]. Mutations within genes have also been proposed to be studied within a matrix as inputs for machine learning methods [40–43]. In our study, we have proposed a novel method, DeepCues. DeepCues utilized the raw sequence as inputs, which by nature integrates all somatic mutations and germline variants, and also INDELs, to be studied as inputs in a joint manner. Convolutional Neural Networks (CNNs) were then applied to train classifiers for cancer type classification. Furthermore, we have included a fully connected layer to allow for relevant gene discovery to help characterize genes and pathways important for multiple cancers.

As a pilot study, we retrieved germline and somatic DNA sequencing data from matched samples across seven types of cancer and used DeepCues to perform cancer type classification. Of note, the COSMIC has combined genome-wide sequencing results from 542,000 tumors with complete manual curation of 23,489 individual publications [32, 44]. Using 985 known pathogenic transcripts as input, we obtained 73.9% and 77.6% accuracy using germline raw sequencing and cancer sequencing data as inputs, respectively. In our results, DeepCues was also found to significantly outperform conventional methods (*p* < 0.01). Consistent with somatic mutations playing a large role in cancer [45], integration of somatic mutations together with germline data significantly improved overall accuracy (*p* = 0.005) using the 985 known pathogenetic transcripts. Integration of somatic data significantly increased accuracy for breast cancer (*p* = 6.6E−03), brain cancer (*p* = 3.8E−06), and uterine cancer (*p* = 1.92E−02), suggesting somatic mutations play a relatively larger role in these cancers. Following the integration of additional 985 unknown transcripts into the model, we were able to boost overall accuracy to 82.7% and 80.0% for germline sequences and cancer sequences, respectively. As an observation, after integrating the additional 985 transcripts, cancer raw sequences were not superior to germline sequencing, suggesting that the addition of somatic mutations was not informative for cancer type prediction. This observation is partially due to the fact that traditional cancer driver gene research’s focus on somatic mutations. As a result, the 985 additional transcripts that were not previously identified as cancer driver genes, are most likely not enriched for cancer relevant somatic mutations. Conventional methods are limited regarding germline variation and their interactive role in cancer due to a large number of variables and complexity issues. In our study, we were able to obtain reasonable accuracy performances using the germline raw sequence only as an input. This suggests that germline variation may be more important than previously reported based on prior methods [34]. More specifically, we found that breast cancer and colorectal cancer have the best performance using only germline information, suggesting that these two cancers probably confer higher heritability compared to others. Studies have reported high familial heritability in breast cancer and colorectal cancer too [46]. Using a fully connected layer in our framework, we identified relevant known and unknown pathogenic genes. For the 20 genes we have identified to be relevant for breast cancer, strikingly, 40% of the genes have been reported in the COSMIC top 20 genes for breast cancer.

## Conclusions

Future development to better evaluate and assess our model will involve the inclusion of gene expression level, copy number variation, methylation, as well as including additional transcripts to be studied. Given that DeepCues is novel in its ability to utilize germline data in an informative manner, it will be of great interest and clinical impact to apply DeepCues to differentiate cancerous and non-cancerous samples. Disease classification not only allows for improved diagnosis and therapies but also allows research to understand a disease through identified groups of genes and related pathways. DeepCues uses genetic sequencing data as inputs with little domain knowledge and feature preparation. With the abundance of genomic information available, we expect DeepCues can be used in a variety of disease settings to help profile diseases.

## Methods

Due to the nature of 64 codons in human genetics, the input layer in component (A) uses one hot encoding to represent each input sequence as a N * 64 binary matrix, where N equals the number of codons. Therefore, the input can be considered as a 1-D sequence with 64 channels. Component (B) is an encoder layer to encode the input to a lower dimensional vector. The encoder component contains a sequence of convolutional layers with six output channels and a fully connected layer for each output channel. Therefore, a vector of six outputs is generated by the encoder for each input sequence. In theory, the output channel can be set as any positive integer. The more output channel, the more expressive capacity and more complexity of the model. To make a trade-off between the complexity and the expressive capacity, we set the output channel as six. In fact, if the precision of each channel is 0.01 (i.e., can store 100 numbers), 6 channels can express ${100}^{6}$ different samples. One convolutional layer is composed of one 1-D convolutional layer followed by a Leaky Rectified Linear Unit (LeakyReLU) as the activation function and an average pooling layer. The number of convolution layers is determined by the transcript length N and the kernel size for average pooling layer. A Kernel size of six was used for the average pooling. Therefore, we will have ${\mathrm{log}}_{6}\mathrm{N}$ convolution layers for each transcript. Component (C) is a fully connected layer with k outputs for k diseases. The inputs of component (C) are the combinations of products from the component (B) generated under the sequence of transcripts. With the average of 3375 bases in the transcripts, the encoder layer would have an average of 3–4 convolution layers. Of note, we set the following parameters for our model: the number of input channels for the encoder layer: 64; the convolution kernel size: 3, the output channel size of the encoder layer: 3; learning rate: 0.001; batch size: 32; number of learning epochs: 30. We used cross entropy loss as the loss function and Adam algorithm as the optimizer.

A training set, validation set, and test set were created by randomly splitting the samples using a 7:1:2 ratio, respectively. Parameters were trained using the training set and tuned using the validation set. Precision, recall, and F-measure were calculated for each cancer type using the testing set. To compare the performance of our models to other conventional methods for cancer classification, we applied penalized logistic regression with L1 penalty, linear support vector machine (SVM), gradient boosting decision tree (GBDT), and Multiple Layer Perceptron (MLP) [14, 47]. Inputs for regression, SVM, GBDT are point mutations, whereas input for MLP are sequence data. The performance was also compared between the germline raw sequence and the cancer raw sequence. For the DeepCues, evaluations were repeated ten times with different initial seeds.

To reduce computational load, we selected genes that have been implicated in cancer using a list of 719 consensus genes (Additional file 1: Table S1) from the Catalogue of Somatic Mutations in Cancer (COSMIC). COSMIC is a mutation catalogue with comprehensive mutation information curated from about 542,000 tumor samples [32]. In our dataset, we found these consensus genes corresponded to 985 transcripts (Additional file 2: Table S2), and we used these transcripts to train and evaluate our proposed classifiers. We compared DeepCues with multiple conventional methods and state-of-the-art method, including penalized logistic regression (L1 penalty), SVM with linear kernel, Gradient Boosting Decision Tree (GBDT), and MLP model. The regression, SVM, and GBDT baseline model was trained using germline variants and somatic mutations found in these selected transcripts. Default parameters were used for the baseline models in scikit-learn (v0.22). To discover potentially relevant genes not known to be implicated in cancer, we also applied a multinomial logistic regression model to the remaining transcripts using disease type as an output, and the number of mutations in each transcript as inputs to identify the 985 top ranked transcripts based on p-value (Additional file 3: Table S3). The inputs for the multinomial are number of mutations in each transcript. The number of mutations has been normalized by gene length. Classifiers were trained, and evaluation was measured using only known pathogenic transcripts and also using a combination of the known and unknown pathogenic transcripts. It has been demonstrated that features frequently ranked high in different training sets yields a robust set of predictive features with stability [48]. To obtain a gene list with reasonable stability, we repeated training the classifiers with random seeds and reported the top 20 most frequent transcripts in each replication. An earlier version of this article was previously published as a preprint [49].

## Supplementary Information

**Additional file 1**. **Table S1**. 719 known oncogenes used in our pilot study

**Additional file 2**. **Table S2**. 985 transcripts corresponding to the 719 genes

**Additional file 3**. **Table S3**. 985 additional transcripts selected for analyses using multimodal logistic regression

**Additional file 4**. **Table S4**. Top genes in the 985 pathogenetic cancer transcripts

**Additional file 5**. **Table S5**. Top genes of the 985 pathogenetic germline transcripts

**Additional file 6**. **Table S6**. Top genes of the 1970 pathogenetic cancer transcripts

**Additional file 7**. **Table S7**. Top genes of the 1970 pathogenetic germline transcripts

## Data Availability

Somatic mutations and aligned sequence files (bam files) for germline variants generation are available upon application to the access controlled TCGA data (https://portal.gdc.cancer.gov) through database of Genotypes and Phenotypes (dbGap) application. The proposal and data application have been approved by dbGap application. All codes necessary to process the sequencing data and to re-generate the results are publicly available at https://github.com/zexian/DeepCues.

## Abbreviations

CNNs: Convolutional neural networks
DeepCues: Deep learning for disease Classification using exome sequencings
TCGA: The Cancer Genome Atlas
COSMIC: Catalogue of somatic mutations in cancer
GBDT: Gradient Boosting Decision Tree
MLP: Multiple Layer Perceptron
LeakyReLU: Leaky Rectified Linear Unit
SVM: Support vector machine
